# Optimized Hydrothermal Pretreatment of Quinoa Seeds and Its Impact on the Functional, Rheological, and Breadmaking Properties of Quinoa Flour

**DOI:** 10.1111/1750-3841.71508

**Published:** 2026-09-25

**Authors:** Olayemi Eyituoyo Dudu, Olawunmi Olumayowa Bolaji, Taiwo Adedola Olotu, Rukayat Abidemi Tajudeen, Jessica Wemimo Dudu, Samson Adeoye Oyeyinka

**Affiliations:** ^1^ Department of Food Science and Technology Bells University of Technology Ota Ogun State Nigeria; ^2^ School of Engineering Sciences, Separation Science Department LUT University Kouvola Finland; ^3^ National Centre for Food Manufacturing University of Lincoln Holbeach UK; ^4^ Centre for Innovative Food Research (CIFR), Department of Biotechnology and Food Technology, Faculty of Science University of Johannesburg Johannesburg Gauteng South Africa

**Keywords:** bread, flour rheology, functional properties, sensory evaluation, wheat flour substitute

## Abstract

In this study, hydrothermal treatment conditions for quinoa flour were optimized using response surface methodology, evaluating conditioning time (6–12 h) and drying temperature (50–90°C) against key quality attributes, namely, swelling power and peak viscosity as swelling/expansion indexes, breakdown viscosity as a thermomechanical stability index, and solubility and setback viscosity as retrogradation tendency indexes. The optimal treatment was identified as 12 h conditioning followed by drying at 50°C, with drying temperature exerting the greatest influence on functional properties. The functional characteristics of the optimized hydrothermally treated quinoa flour (OHQF) were subsequently evaluated. Compared with untreated flour, OHQF exhibited increased water absorption capacity (6%), oil absorption capacity (30%), and bulk density (25%), while solubility (33%), peak viscosity (10%), and setback viscosity (4%) were reduced. These changes indicate improved hydration behavior and reduced retrogradation potential. The effects of substituting wheat flour with OHQF at 10%, 20%, and 30% (w/w) on dough rheology and bread quality were assessed, using composite flours containing untreated quinoa flour as controls. At all substitution levels, OHQF increased farinograph water absorption, dough development time, and dough stability and enhanced extensograph dough resistance to extension at 90 min while maintaining improved dough extensibility. Although bread loaf volume decreased with OHQF incorporation, sensory evaluation showed improved acceptability, particularly in crust color and appearance, regardless of substitution level. Overall, 10% substitution with OHQF produced bread with properties most comparable to 100% wheat flour. These findings demonstrate the potential of hydrothermally treated quinoa flour as a functional ingredient and are of direct relevance to the flour milling and baking industries.

## 1 Introduction

The over‐reliance on wheat flour is a major challenge to African countries, as it contributes to high import bills and exposes food systems to global supply chain disruptions. Thus, it is imperative to utilize indigenously cultivated cereals with similar flour properties to those of wheat flour for the production of baked foods, which are major staples in this region.

Quinoa (*Chenopodium quinoa*) is a pseudocereal with an exceptional ability to thrive in different climatic conditions (Pedrali et al. 2023; Ramos‐Tarifa et al. 2026). It is a pale‐yellow seed, which occasionally deviates from white, pink, and red to black depending on the variety, and it has a bland taste, which gives it a competitive advantage (Huang et al. 2025). Quinoa is regarded as a high‐protein (≈10‐18%) seed, which contains all essential amino acids, as well as is rich in fiber, vitamins, minerals, and bioactive compounds (Ramos‐Pacheco et al. 2025; Guardianelli et al. 2022; Ng and Wang 2021; Mu et al. 2023). These nutritional benefits have been exploited in food systems; notably, quinoa flour, which is gluten‐free, has been utilized in the production of different baked foods such as pancakes (Vejdanivahid and Salehi 2025), cookies, bread (Cotovanu et al. 2023), and snacks (Baskaya‐Sezer 2025). However, similar to other gluten‐free flours, quinoa flour has poor compatibility with gluten in wheat flour, which results in altered rheological properties and baked foods with compromised structures when combined (Mu et al. 2023). Thus, improving the compatibility of wheat and quinoa flours may facilitate the development of nutritionally enhanced wheat‐based baked products. Quinoa contributes high‐quality protein, dietary fiber, essential minerals, and bioactive compounds that can improve the nutritional profile of composite flour products. Furthermore, in regions where quinoa is widely cultivated and wheat is largely imported, the partial substitution of wheat flour with quinoa flour may reduce reliance on imported wheat while supporting the production of value‐added bakery products.

Preprocessing cereal grains before milling has the potential of alleviating the shortcomings of gluten‐free cereal flours. Particularly, hydrothermal pretreatment conditions involving conditioning of cereal grains to enhance hydration of their endosperm, followed by drying and milling of the grain, have been reported to have beneficial effects on flour yield, particle size, and breadmaking properties (Jemal and Awulachew 2025; Naraharasetti et al. 2025). The combination of conditioning and drying for the hydrothermal pretreatment of cereal grains represents a low‐cost, chemical‐free approach that has the potential to modify grain constituents to enhance subsequent flour functionality. Such treatment has the potential of inducing structural changes in starch and protein matrices of flour, thereby improving baking performance (Van Rooyen et al. 2022). Despite established applications in conventional cereals such as wheat, sorghum, millet, etc., the systematic optimization of conditioning and drying variables for the hydrothermal pretreatment of quinoa remains largely unexplored. Additionally, the subsequent effect of such hydrothermally pretreated quinoa flour on the rheological and breadmaking properties of wheat flour in a composite flour system remains to be studied. The application of such hydrothermal pretreatment on quinoa flour may enhance its structural synergy with wheat gluten in producing well‐structured leavened bread.

In this study, quinoa seeds were subjected to hydrothermal pretreatment consisting of a combination of different conditioning times and drying temperature variables. The combined effect of the hydrothermal pretreatment variables (HPVs) on the functional properties of quinoa flour was studied. Subsequently, the HPVs were optimized to produce flour properties tailored for bread making. Furthermore, the effect of the optimized hydrothermally pretreated quinoa flour (OHQF) on the rheological and breadmaking properties of wheat flour in a composite flour system was studied.

## 2 Materials and Methods

### 2.1 Materials

Quinoa seeds (12.96% protein) were purchased from a grain market in Jos, Plateau State, Nigeria. Major bread ingredients such as all‐purpose wheat flour (11.92% protein, Mamagold, Supreme flour mills), margarine (Devon King's, PZ Wilmar), instant dry yeast (STK Royal, Guangxi Danbaoli Yeast Co. Ltd), sugar (Dangote, Dangote), and salt (Mr. Chef, Royal Salt Limited) were purchased from Ota main market, Ota, Ogun State, Nigeria. All reagents used in this study were of analytical grade.

### 2.2 Hydrothermal Treatment of Quinoa Flour

Quinoa seeds were sorted and dry‐sieved to remove sand, stones, and other residual particulate matter. The seeds were further steeped in potable tap water for 10 min for residual particulate matter to settle at the base of the steeping container. The seeds were separated from the impurities and then washed with potable tap water, air‐dried for 30 min, and dried in a convection oven (DHG‐9240A, China) at 45°C for 6 h, which achieved a 10.12 ± 0.02% moisture content. The seeds (200 g) were conditioned in deionized water at room temperature (25 ± 2°C) for 6–12 h. The seeds were thoroughly drained and dried at temperatures ranging from 50 to 90°C for an average of 6 h. The hydrothermally treated quinoa seeds were milled, passed through a 200‐µm sieve, and stored in air‐tight bags for further analysis.

### 2.3 Water and Oil Absorption Capacity

Water and oil absorption capacities of untreated and hydrothermally treated quinoa flours were quantified using the protocol of Poshadri et al. (2023). A 10% w/v suspension of 1 g of flour and 10 mL of deionized water or sunflower oil was weighed into a clean and dried centrifuge tube. The suspension was vortexed and centrifuged at 2000 g for 30 min using a benchtop centrifuge (B06322, Allegra X‐30 Series, Beckman Coulter, USA), and the suspended phase was discarded. Water and oil absorption capacity was calculated as the weight in g/g of the water or oil absorbed in the flour relative to the dry flour sample.

### 2.4 Swelling Power and Solubility

Swelling power and solubility of untreated and hydrothermal‐treated quinoa flours were measured using the method described by Medha et al. (2026) with some modifications. A 1% w/v suspension consisting of 0.1 g of flour and 10 mL of deionized water was weighed into a clean and dried centrifuge tube. The suspension was vortexed and centrifuged at 3500 ×g for 20 min using a benchtop centrifuge (B06322, Allegra X‐30 Series, Beckman Coulter, USA). The suspended phase was poured into dried moisture cans and then oven‐dried at 105°C for 1 h, and the residue in the can was weighed. The gel at the base of the tube was also weighed. Swelling power and solubility were calculated as shown below:

$$\mathrm{Swelling}\;\mathrm{power}\;\;\left(\frac{g}{g}\right)=\frac{W_{\mathrm{gel}}}{W_{\mathrm{sample}}-W_{\mathrm{residue}}}$$

$$\mathrm{Solubility}\;\left(\%\right)=\frac{W_{\mathrm{residue}}}{W_{\mathrm{sample}}}\times \;100$$

Where *W*_sample_ is the weight of the untreated and all treated quinoa flours, *W*_g_ is the weight of gel, and *W*_residue_ is the residue from the dried and suspended phase.

### 2.5 Bulk Density

The tapped bulk density of untreated and hydrothermal‐treated quinoa flour was determined following the method described by Charles et al. (2026) with slight modification. Flour (5 g) was weighed into a 50 mL measuring cylinder and tapped lightly on a workbench until no drop in volume was observed. Tapped bulk density was calculated as the weight per volume of tapped flour in g/mL.

### 2.6 Pasting Properties

Pasting properties of untreated and hydrothermal‐treated quinoa flours were determined by a rapid visco‐analyser (RVA, 2122833, PerkinElmer, Perten Instruments, Sweden). The protocol used was that described by Dudu et al. (2024). A 14% w/v suspension of 3.5 g of flour and 25 mL distilled water was inserted into an RVA canister, and an RVA spindle was inserted into the suspension. The RVA experiment was carried out using these parameters: mixing for 1 min, heating to 50°C within 1 min, then to 95°C within 3.5 min, holding the temperature at 95°C for 2.5 min, cooling to 50°C in 4 min, and holding the temperature at 50°C for 2 min. Pasting characteristics data of peak viscosity, breakdown viscosity, and setback viscosity were extracted from the RVA using the Newport Thermocline version 3 for Windows software.

### 2.7 Farinograph Properties of Quinoa‐Wheat Flours

The farinograph properties of untreated and optimally hydrothermally treated quinoa flour blended with wheat flour at 10:90, 20:80, and 30:70 w/w were determined using a Farinograph (TS‐816100‐001, Brabender GmbH & Co., Germany) in accordance with a modified AACC international method 54‐21. Briefly, wheat flour or composite flour (300 g, 14% moisture) was mixed with water, which was gradually added from a burette, and concurrently, a graph was plotted. The following data were derived: water absorption, which is the amount of water required to center the farinograph curve between 480 and 520 BU. Other properties such as dough development time, maximum consistency, stability, and mixing tolerance index of the flour were extrapolated from the Farinograph‐TS software.

### 2.8 Extensograph Properties of Quinoa‐Wheat Flours

The extensograph properties of untreated and optimally hydrothermally treated quinoa flour blended with wheat flour at 10:90, 20:80, and 30:70 w/w were analyzed using an Extensograph (TS‐860704, Brabender GmbH & Co., Germany) in accordance with AACC International Method 54‐10.01. A precise flour quantity was weighed on a 14% moisture basis, combined with 6 g of salt, and water was added to the flour based on farinograph absorption data for each flour mixture. Dough mixing followed, which was done according to the time range from 2 to 6 min using the Farinograph. The dough produced (150 g) was shaped into a ball, then into a cylinder shape for simulation using a mold, fixed, and allowed to rest in a fermentation chamber at 30°C for 45 min. The experiment was repeated at 90 and 135 min of resting times. After resting, the dough was clamped in a holding device. The hook stretched the dough downward while the extensograph plotted a graph in which resistance to extension, extensibility, and area data were extrapolated after 5 mm of the base of the plot was attained.

### 2.9 Bread‐Making Method

Optimized condition treated quinoa flour was blended with wheat flour at ratios of 10:90, 20:80, and 30:70 w/w. The composite flours were utilized in producing pan bread using a straight dough method. The standard recipe was used for the bread formulation, consisting of flour (1000 g), water (according to the farinograph water absorption requirement presented in Table 5), yeast (10 g), sugar (120 g), margarine (10 g), and salt (20 g). All ingredients, including water (at 25°C), were mixed into dough using a spiral mixer (DIOSNA, SP24‐120). The dough was divided into four portions (350 g each) and molded using a dough molder (MACADAMS). The molded dough was placed in a lightly greased pan and then proofed in a proofing chamber (WACHTEL, Aeromat) at 90% relative humidity and 35°C. The proofed dough was baked in an oven (MIWE) at 180°C for 30 min. The bread was cooled to ambient temperature and stored in airtight nylons at ambient conditions for further analysis. Bread containing only wheat flour and those containing untreated quinoa flour were considered as controls.

### 2.10 Loaf Volume

Bread loaf volume data was determined using a volume analyzer (BVM‐L370, TEXVOL instruments). A rod is placed at the center of the bread loaf, which acts as support. A rotating arm equipped with a laser moves in a half circle around the bread to capture its volume. Bread loaf volume data was extrapolated from the analyzer using vocal software (TEXVOL instruments).

### 2.11 Sensory Evaluation

A panel of 105 semi‐trained panelists who are regular consumers of bread was utilized for the evaluation, comprising males and females with an average age of 20 years from Bells University of Technology. People who had known dietary issues with the consumption of wheat bread did not partake in the evaluation. A Multiple Comparison test method using a 9‐point hedonic scale rating where point “9” indicated extreme likeness and point “1” indicated extreme dislike was used to investigate the sensory properties of wheat flour bread containing untreated or optimally treated quinoa flour. Bread samples were coded with three random characters and presented to each panelist in random order. Each panelist was requested based on a questionnaire to compare each wheat flour bread containing untreated and optimized hydrothermally treated quinoa flour against 100% wheat flour bread in terms of crust color, appearance, aroma, taste, mouthfeel, crumb texture, and overall acceptability.

### 2.12 Optimization of Hydrothermal Treatment Variables

Hydrothermal variables for the treatment of quinoa flour were optimized using response surface methodology. A two‐variable central composite design with two independent variables, namely, conditioning time (X1) and drying temperature (X2), was examined in relation to dependent variables: peak viscosity, breakdown viscosity, setback viscosity, and pasting temperature, respectively, using Design Expert (DExp) software (version 13, Stat Ease Inc., Minneapolis, MN, USA). Conditioning time and drying temperature conditions for quinoa flour were derived from preliminary investigations. These conditions were computed into DExp software. Experimental conditions were generated for each independent variable and coded as −1, 0, and 1 by DE.

Consequent conditions were utilized in studying the effect of the independent variables in relation to the dependent variables. Each dependent variable was fitted into a second‐order quadratic regression model, which is in Equation 1:

(1) $$Y=\beta_{0}+\beta_{1}C_{t}+\beta_{2}D_{t}+\beta_{11}C_{t}D_{t}+\beta_{21}{C_{t}}^{2}+\beta_{22}{D_{t}}^{2}$$

where 𝑌 is the response function, *β_0_* is the intercept, *β_1_* and *β_2_* are the linear coefficients, *β_11_* is the interactive coefficient, *β_21_* and *β_22_* are the quadratic or squared coefficients, C*_t_* is conditioning time, and *D_t_* is drying temperature. Analysis of variance (ANOVA) at a significance level of 0.05 was used to examine the regression equations. In determining the optimized factor conditions, a numerical technique was used. In determining the optimized malting conditions, only response models with a significant P‐value (P ≤ 0.05) and a high coefficient of regression (*R* ^2^) value were considered for numerical optimization. It is important to mention that the overall objective of this study is to enhance the bread‐making performance of quinoa flour. In achieving this, the modified quinoa flour should elicit higher swelling, expansion capacity, and thermal stability as well as a lower retrogradation tendency, which is related to the texture of bread, than its untreated form. Based on this, swelling power and peak viscosity were used as indexes of swelling and expansion capacity, breakdown viscosity was used to predict the thermal stability and solubility, and setback viscosity was used as an index of retrogradation tendency of the flour. To this end, the numerical optimization entailed keeping the independent variables within their experimental ranges, while decisions were made to maximize swelling power and peak viscosity and to minimize breakdown viscosity, solubility, and setback viscosity. These decisions were processed by a desirability function (Equation 2) (Myers et al. 2016):

(2) $$D(x)=(d_{1}\;\times \;d_{2}\;\times \;\text{…}..\;d_{n})1/n$$

where *D*(x) is the desirability function, *d_1_*, *d_2_*, and *d_n_* are the desirability indices for each response variable, and *n* is the number of considered dependent variables. The desirability function generates a combination of optimized parameters and assigns scores that range from 0 (undesirable) to 1 (most desirable). The conditioning time and drying temperature conditions were chosen from those suggested by the DExp software. The predicted optimized conditions were validated through laboratory experiments.

### 2.13 Statistical Analysis

All experiments were conducted at least three times, and data were presented as mean ± standard deviation. Statistical analyses were performed using Statistical Package for Social Sciences (SPSS, version 26, IBM, Chicago, USA). Differences between the two samples (QF and OHQF) were evaluated using Student's *t*‐test, and significance was established at *P* ≤ 0.05. For datasets involving more than two groups, one‐way analysis of variance (ANOVA) was used, followed by Duncan's Multiple Range test for mean separation when significant differences were detected.

## 3 Results and Discussion

### 3.1 Model Description

The investigated quality characteristics of hydrothermal‐treated quinoa flour are shown in Table 1. The selection of these attributes was based on their impact on baking flour. The maximum paste viscosity achieved under heating and shearing conditions, known as peak viscosity, was used to forecast how well quinoa flour will expand after baking. The thermal stability of quinoa flour during baking was predicted using breakdown viscosity, which is the difference between peak and holding viscosities of paste under heating and shearing circumstances. The ability of the flour's paste to retrograde after cooling was predicted using setback viscosity, which is the difference between the final viscosity of cold paste and the holding viscosity of hot paste under heating and shearing conditions.

**TABLE 1:** Central composite design for hydrothermal condition treatment of quinoa flour.

Experimental runs	Conditioning time	Drying temperature	Swelling power	Solubility	Peak viscosity	Breakdown viscosity	Setback viscosity
	(h)	(°C)	(g/g)	(%)	(cP)	(cP)	(cP)
1	12	50	12.33	7.80	1861	374	1752
2	6	50	12.25	8.90	2122	528	2191
3	9	70	11.31	13	1547	154	1673
4	9	70	11.39	15	1547	156	1668
5	9	41.72	12.23	10.30	1815	281	2010
6	12	90	11.26	13.10	1815	276	2015
7	9	70	11.30	13	1547	154	1673
8	4.76	70	11.58	19.40	1557	179	1452
9	9	98.28	11.39	10.10	1019	42	1220
10	9	70	11.50	9	1547	151	1678
11	9	70	11.31	13	1555	157	1679
12	6	90	11.11	14	1357	130	1311
13	13.24	70	11.19	8.30	1678	287	1436

**FIGURE 1:**
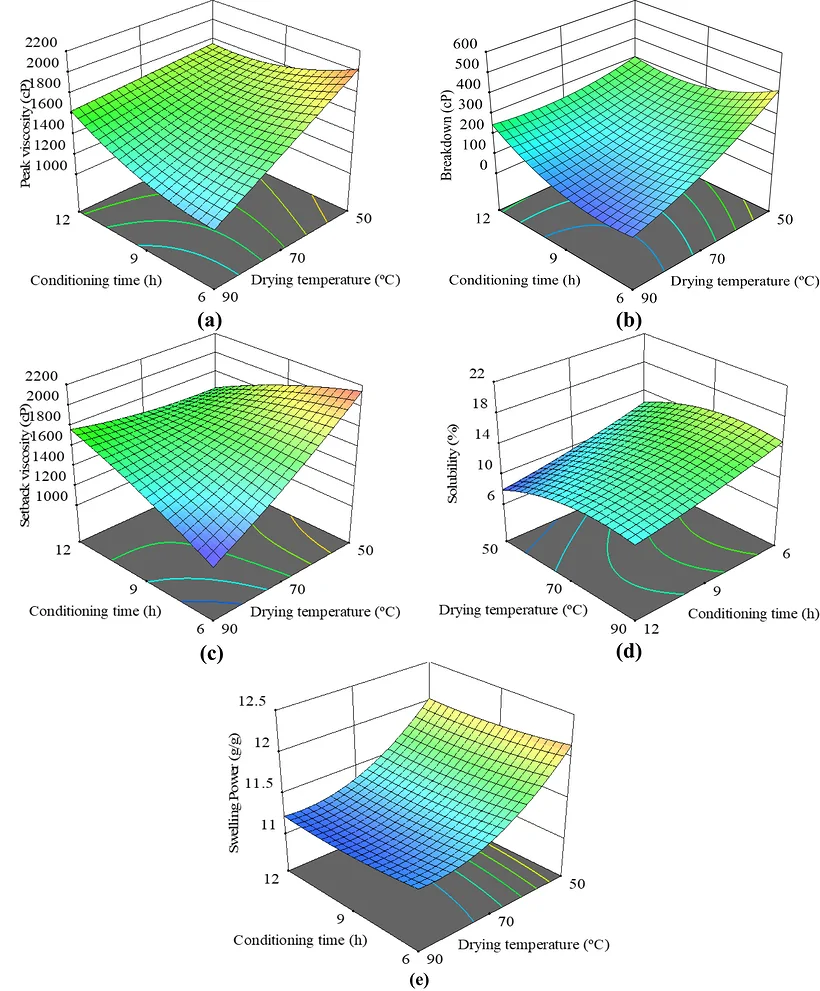
a: Response surface plot showing the effect of hydrothermal pretreatment conditions (conditioning time and drying temperature) on peak viscosity; b: Response surface plot showing the effect of hydrothermal pretreatment conditions (conditioning time and drying temperature) on breakdown viscosity; c: Response surface plot showing the effect of hydrothermal pretreatment conditions (conditioning time and drying temperature) on setback viscosity; d: Response surface plot showing the effect of hydrothermal pretreatment conditions (conditioning time and drying temperature) on solubility; e: Response surface plot showing the effect of hydrothermal pretreatment conditions (conditioning time and drying temperature) on swelling power.

Data in Table 1, which consisted of independent variables (conditioning time and drying temperature) and dependent variables, were fitted into a second‐order regression model for each response variable shown in Table 2. The ANOVA of each model is shown in Table 3. Regression coefficients (*R* ^2^) data indicated that 54% to 87% of the total variations in the data were explained by the models. Additionally, the P‐values of all the models ranged from 0.01 to 0.27, indicating that they were reliable as predictive models, with the exception of the solubility model. Corresponding 3D surface plots for each model are shown in Figure 1.

**TABLE 2:** Regression coefficient table indicating the response models.

Coefficients	Peak Viscosity	Breakdown Viscosity	Setback Viscosity	Swelling Power	Solubility
*β_0_*	1548.60	154.20	1674.20	11.36	12.60
C_t_	45.95	18.15	30.38	−0.04	−2.21
D_t_	−242.03	−104.19	−216.78	−0.42	1.26
C_t_ D_t_	179.88	75.13	285.75	0.02	0.05
C_t_^2^	102.29	71.74	−43.26	0.05	0.36
D_t_^2^	2.04	35.99	42.37	0.26	−1.47

*Note*: *β_0_* is intercept, C_t_ is Conditioning time, D_t_ is drying temperature.

**TABLE 3:** Analysis of variance (ANOVA) of the response models.

Source of variance	df	Peak viscosity		Breakdown viscosity		Setback viscosity		Swelling power		Solubility	
		F‐value	p‐value	F‐value	p‐value	F‐value	p‐value	F‐value	p‐value	F‐value	p‐value
C_t_	1	0.74	0.42	0.46	0.52	0.25	0.63	0.29	0.61	4.63	0.07
D_t_	1	20.52	0.003*	15.27	0.01	12.69	0.001*	36.65	0.001*	1.51	0.26
C_t_D_t_	1	5.67	0.05*	3.97	0.09	11.02	0.01*	0.03*	0.87	0.001*	0.97
C_t_^2^	1	3.19	0.12	6.30	0.04*	0.44	0.53	0.39	0.55	0.10	0.76
D_t_^2^	1	0.001*	0.97	1.58	0.25	0.42	0.54	11.86	0.01*	1.77	0.22
Model	5	6.03	0.02*	5.38	0.02*	4.99	0.03*	9.77	0.005	1.63	0.27
Lack of fit		4161.18	0.20	2378.84	0.05	3507.83	0.12	10.90	0.02	2.78	0.17
*R* ^2^		0.81		0.79		0.78		0.87		0.54	

*Note*: *indicate significant factors (p ≤ 0.05), df is degree of freedom, C_t_ is Conditioning time, D_t_ is drying temperature and *R* ^2^ is adjusted *R* ^2^.

#### 3.1.1 Peak Viscosity Model

Peak viscosity is the measure of the highest viscosity attained by a starch system, which is related to its swelling and expansion properties (Iuga and Mironeasa 2019). The peak viscosity model had a p‐value of 0.02 and *R* ^2^, which indicated that 19% of the total variation could not be explained by the model (Table 3). Notably, significant effects on peak viscosity were induced by the linear effect of drying temperature and the interactive effect of conditioning time and drying temperature. This was buttressed by the response surface plot, which revealed that a decrease in drying temperature and conditioning time led to an increase in peak viscosity (Figure 1a). While an increase in drying temperature and a decrease in conditioning decreased peak viscosity. Thus, the peak viscosity model had a significant p‐value (P ≤ 0.05) and a high *R* ^2^, which implied that it would be suitable as a predictive model.

#### 3.1.2 Breakdown Viscosity Model

Breakdown viscosity is the measure of the thermo‐mechanical stability of a starch‐based paste. The breakdown viscosity model had a p‐value of 0.02 and *R* ^2^, which indicated that 21% of the total variation could not be explained by the model (Table 3). Notably, significant effects on breakdown viscosity were induced by the linear effect of drying temperature and the interactive effect of conditioning time and drying temperature, including the quadratic effect of conditioning time. This was buttressed by the response surface plot, which revealed that a decrease in drying temperature and conditioning time led to an increase in breakdown viscosity (Figure 1b). While an increase in drying temperature and a decrease in conditioning decreased breakdown viscosity, which was similar to the trend elicited by the breakdown viscosity plot. Thus, the breakdown viscosity model had a significant p‐value (P ≤ 0.05) and a high *R* ^2^, which implied that it would be suitable as a predictive model.

#### 3.1.3 Setback Viscosity Model

Setback viscosity is the measure of the retrogradation tendency of a starch‐based paste on cooling. The setback viscosity model had a P‐value of 0.03 and *R* ^2^, which indicated that 22% of the total variation could not be explained by the model (Table 3). Notably, significant effects on setback viscosity were induced by the linear effect of drying temperature and the interactive effect of conditioning time and drying temperature. This was buttressed by the response surface plot, which revealed that an ncrease in drying temperature and decrease in conditioning decreased setback viscosity (Figure 1c), which was similar to the trend elicited by the breakdown viscosity plot (Figure 1b). While a decrease in drying temperature and conditioning time led to an increase in setback viscosity. Thus, the setback viscosity model had a significant P‐value (P ≤ 0.05) and a high *R* ^2^, which implied that it would be suitable as a predictive model.

#### 3.1.4 Swelling Power Model

Swelling power is the measure of both intra‐ and inter‐granular water in a starch system, which is related to the water uptake ability of amylopectin (Iuga and Mironeasa 2019). The swelling power model had a p‐value of 0.01 and *R* ^2^, which indicated that 13% of the total variation could not be explained by the model (Table 3). Notably, significant effects on swelling power are induced by both linear and quadratic effects of drying temperature. This was buttressed by the response surface plot, which revealed that a decrease in drying temperature, regardless of conditioning time, increased swelling power (Figure 1). Thus, the swelling power model had a significant P‐value (P ≤ 0.05) and a high *R* ^2^, which implied that it would be suitable as a predictive model. In the next section, the solubility model was explained.

#### 3.1.5 Solubility Model

Solubility is an index of amylose interaction with water under high hydrothermal conditions, which is critical to the retrogradation ability of baked foods. The solubility model had a P‐value of 0.27 and *R* ^2^, which indicated that 46% of the total variation could not be explained by the model (Table 3). Furthermore, the response surface plot revealed a somewhat dependence on drying temperature; that is, a decrease in solubility was noted at low drying temperature (Figure 1d). Thus, the solubility model had a non‐significant p‐value (P > 0.05) and a low *R* ^2^, which implied that it would not be suitable as a predictive model.

The adequacy of the fitted models was further assessed using lack‐of‐fit tests (Table 3). The peak viscosity, setback viscosity, and solubility models exhibited non‐significant lack‐of‐fit values (p = 0.20, 0.12, and 0.17, respectively), indicating satisfactory agreement between experimental and predicted values. The breakdown viscosity model showed a borderline lack‐of‐fit value (p = 0.05), while the swelling power model exhibited a significant lack‐of‐fit (p = 0.02). Nevertheless, the swelling power model was retained due to its high coefficient of determination (*R* ^2^ = 0.87) and significant model terms associated with drying temperature. For the peak viscosity model, the *R* ^2^ value of 0.81 indicates that 81% of the observed variation was explained by the model, while the remaining 19% represents residual variance. This unexplained variation may be attributable to factors not included in the model, such as heterogeneity in quinoa seed composition, variations in moisture redistribution during conditioning, structural differences in starch granules, and experimental variability associated with milling and RVA measurements. Despite this residual variance, the non‐significant lack‐of‐fit result supports the adequacy of the model for prediction within the investigated experimental domain.

### 3.2 Optimal Hydrothermal Conditions for Quinoa Flour

Based on the ANOVA data, all models apart from the solubility model were used for numerical optimization. In achieving quinoa flour with high swelling/expansion property and thermal stability, including low retrogradation tendency, the DE software was instructed to maximize peak viscosity and swelling power and to minimize breakdown viscosity and setback viscosity. The resulting optimal condition was derived as 12 h conditioning time and 50°C drying temperature with a desirability index of 0.66. A validation experiment was done, and the standard deviations were no more than ±0.1 from the predicted independent variables. Although breakdown viscosity was specified as a response to be minimized, the selected optimum exhibited a higher breakdown viscosity than the untreated flour. This outcome reflects the multi‐response nature of the desirability optimization, whereby the selected condition represented the best compromise among competing objectives. The desirability value of 0.66 indicates that some responses, including breakdown viscosity, were not fully optimized individually but were accepted to achieve superior overall functional performance.

### 3.3 Techno‐Functional Properties of Optimized Hydrothermal‐Treated Quinoa Flour

#### 3.3.1 Water and Oil Absorption Capacities

Water and oil absorption capacities are crucial in defining the textural and organoleptic properties of food, especially in baked goods. The optimal hydrothermal treatment conditions improved the water and oil absorption capacities of quinoa flour by 6% and 30%, respectively (Table 4). This suggests that the hydrothermal treatment induced some conformation changes to the flour by increasing more hydrophilic and lipophilic zones on its granule surfaces, as well as increasing the binding ability of its protein functional groups to lipophilic compounds (Hasmadi et al. 2020; Kakar et al. 2022). The increase in both hydration properties may be advantageous in baked food applications by impacting dough consistency, flavor retention, and mouthfeel properties (Kumari et al. 2024). The increased water absorption capacity may be attributed to moisture‐induced rearrangement of starch and protein components during hydrothermal treatment (Dutta et al. 2015). Partial protein denaturation can expose additional polar amino acid residues capable of binding water, while localized disruption of starch granule organization may create new sites for water immobilization (Malik et al. 2021; Prashanth et al. 2024). Similarly, the increase in oil absorption capacity suggests exposure of hydrophobic protein regions following thermal treatment, enhancing interactions with non‐polar lipid molecules. These structural modifications are beneficial in bread systems because increased water retention supports dough hydration and handling properties, whereas improved oil binding contributes to flavor retention, crumb softness, and overall eating quality.

**TABLE 4:** Functional properties of untreated and optimal condition treated quinoa flour.

	QF	OHQF
WAC (g/g)	1.21 ± 0.04^a^	1.29 ± 0.05^a^
OAC (g/g)	1.31 ± 0.01^b^	1.70 ± 0.02^a^
Swelling power (g/g)	11.89 ± 0.22^a^	12.04 ± 0.13^a^
Solubility (%)	11.70 ± 0.12^a^	7.90 ± 0.04^b^
Bulk density (g/mL)	0.83 ± 0.01^a^	1.04 ± 0.03^a^
Peak viscosity (cP)	1962.00 ± 1.05^a^	1761.00 ± 1.56^b^
Breakdown viscosity (cP)	277.00 ± 1.27^b^	309.00 ± 1.03^a^
Setback viscosity (cP)	1706.00 ± 1.12^a^	1634.00 ± 1.52^b^
Pasting temperature (°C)	78.28 ± 0.04^b^	83.32 ± 0.07^a^

Values are presented as mean ± standard deviation (SD). Means within a row bearing different superscript letters are significantly different according to Student's *t*‐test at *P* < 0.05.

*Note*: QF is native quinoa flour, OHQF is optimized condition treated quinoa flour, WAC is water absorption capacity, and OAC is oil absorption capacity.

#### 3.3.2 Swelling Power and Solubility

Swelling power and solubility are significant hydration indicators, especially under high thermal processing conditions. Following optimal condition treatment, the swelling power of quinoa flour increased by 1%, while its solubility decreased by 32% (Table 4). This indicates that the treatment prevented amylose leaching from the flour, possibly due to strengthened amylose‐amylopectin, amylose‐amylose, amylose‐protein, and/or amylopectin‐lipid interactions (Subroto et al. 2022). Moreover, hydrothermal treatment of protein‐rich grains such as millet has been reported to induce amino acids to form a spatial arrangement on the surface of their flour/starch granules, which hinders hydration and swelling (Deka et al. 2023). The implication of reduced solubility for breadmaking is that this may lessen excessive starch degradation during baking and contribute to improved crumb stability.

#### 3.3.3 Bulk Density

Bulk density of flour influences packaging efficiency and the structure of baked foods. The bulk density of quinoa flour increased by 25% following optimal condition treatment (Table 4). This may be due to changes in particle structure and moisture retention, resulting in tighter packing of flour granules (Forsido et al. 2021). Moreover, an increase in granule bulk density after hydrothermal treatment has been attributed to partial gelatinization and retrogradation of the granules, thus leading to larger particle size and denser structures (Rozendo et al. 2026).

#### 3.3.4 Pasting Properties

Pasting properties are critical indicators for predicting the swelling/expansion behavior, thermomechanical stability, and retrogradation tendency of starch‐containing systems. The optimal hydrothermal treatment increased pasting temperature and breakdown viscosity by 6% and 12%, while decreasing peak viscosity and setback viscosity by 10% and 4% (Table 4). This phenomenon indicates that the treatment decreased the swelling ability, retrogradation tendency, and thermomechanical stability of quinoa flour. The reduction in peak viscosity following hydrothermal treatment suggests restricted swelling of starch granules. This behavior is often associated with partial gelatinization and molecular reorganization occurring during hydrothermal processing, which results in stronger internal bonding within starch granules. Furthermore, denatured quinoa proteins may interact with starch surfaces to form a physical barrier that limits water penetration and granule expansion during heating. The lower setback viscosity indicates reduced amylose reassociation during cooling, suggesting a lower tendency for retrogradation. From a bread quality perspective, reduced retrogradation is advantageous because it may delay crumb firming and staling during storage (Lončarić et al. 2026). A similar phenomenon was also reported after proso millet was subjected to hydrothermal treatments of blanching and pressure cooking (Javed et al. 2025). The increase in breakdown viscosity indicates a reduction in paste stability because a greater viscosity loss occurred during heating. Under these conditions, starch granules likely retained a greater capacity for water absorption and swelling during heating. While enhanced swelling contributes to improved hydration behavior, it also renders the swollen granules more susceptible to rupture and disintegration under continuous thermal and mechanical stress during RVA analysis. Consequently, a greater viscosity loss occurred between peak and holding stages, resulting in increased breakdown viscosity. This observation highlights the trade‐off between granule swelling and paste stability and explains why the optimization procedure selected a condition with moderate breakdown viscosity in order to achieve a more favorable overall combination of swelling power, peak viscosity, and setback viscosity. Moreover, the hindrance of amylose leaching by the treatment (decreased solubility) implied that there were minimal amounts of leached amylose, which were able to reassociate with amylopectin during cooling of the paste. Furthermore, the increase in pasting temperature signified that the treatment strengthened interactions within the granule structures, thereby creating rigid double helices, which were able to resist disruption by heat. This implied that a higher temperature was required for total destruction of the granule structures (Kunyanee and Luangsakul 2022; Dudu et al. 2024).

### 3.4 Rheological Properties of Optimal Hydrothermally Treated Quinoa Flour in Wheat Flour Matrix

#### 3.4.1 Farinograph Properties

Inclusion of native and hydrothermally treated quinoa flour had contrasting effects on the farinograph properties of wheat flour (Table 5). Corresponding farinograph curves are in Figure S1. Notably, compared with the native quinoa flour, inclusion of the treated flour led to higher water absorption capacity, which had a decreasing trend with an increase in inclusion, though the modified flour had a non‐significant increase in WAC (Table 3). The latter finding is contrary to previous studies, which reported an increase in water absorption with an increase in quinoa flour inclusion (Akturfan and Yalçın 2025; Ma et al. 2022). These studies suggested the increase in water absorption was due to the high protein content of quinoa. The inclusion of quinoa flour elongated the dough development time of wheat flour, which was more prominent at the 30% inclusion level. Compared with the native quinoa flour, inclusion of the treated flour at 10% and 20% levels elicited higher dough development times than their native counterparts. Moawad et al. (2018) reported an increase in dough development time of wheat flour with an increase in quinoa substitution. However, inclusion of quinoa flour had contrasting effects on the maximum consistency of wheat flour. The native flour across all inclusion levels increased the maximum consistency of wheat flour, with 20% inclusion having the highest increase of 5%. Whereas the treated flours across all levels minimally decreased the maximum consistency of wheat flour and were lower than those of their native counterparts. Furthermore, there was a significant increase in dough stability of wheat flour by 6.2 min on 30% quinoa flour inclusion, which indicates enhancement in gluten‐starch networking (Sreechithra et al. 2024). The major effects on the mixing tolerance index of wheat flour were induced by 10% native quinoa flour inclusion, which increased its index by 61%, and the 20% treated quinoa flour, which decreased its index by 65%. An increase in the mixing tolerance index implies that the dough will require a longer kneading period to attain adequate gluten‐starch networking (Shetti 2023). Whereas, a decrease in the mixing tolerance index may be advantageous in producing bread with higher volume (Sreechithra et al. 2024). The improved water absorption and dough development time observed in flours containing OHQF may be linked to the enhanced hydration properties of the treated flour. Hydrothermal treatment likely modified starch and protein structures, increasing their affinity for water and consequently requiring longer hydration periods before optimum dough development was achieved. In addition, interactions between treated quinoa proteins and wheat gluten may have altered the development of the gluten network, resulting in greater dough stability. The increased stability suggests that hydrothermally treated quinoa flour contributed to the formation of a more resilient dough structure capable of withstanding prolonged mixing.

**TABLE 5:** Effect of different substitution levels of optimal hydrothermally treated quinoa flour on the farinograph properties of wheat flour.

Samples	WA (%)	DDT (min)	MC (FU)	DS (min)	MTI (FU)
WF	62.90 ± 0.15^a^	1.48 ± 0.01^d^	497 ± 2^bc^	2.4 ± 0.03^c^	57 ± 0.23^c^
WQF‐10	61.30 ± 0.20^b^	1.40 ± 0.02^d^	502 ± 3^b^	1.60 ± 0.02^d^	92 ± 0.17^a^
WOHQF‐10	62.70 ± 0.17^a^	2.3 ± 0.01^c^	495 ± 1^c^	2.6 ± 0.02^c^	44 ± 0.30^d^
WQF‐20	60.80 ± 0.11^c^	1.50 ± 0.02^d^	520 ± 2^a^	4.4 ± 0.02^b^	51 ± 0.42^c^
WOHQF‐20	62.30 ± 0.10^a^	2.30 ± 0.02^c^	492 ± 3^c^	3.0 ± 0.02^c^	64 ± 0.12^b^
WQF‐30	59.20 ± 0.21^d^	8.30 ± 0.05^a^	507 ± 2^b^	8.6 ± 0.02^a^	64 ± 0.12^b^
WOHQF‐30	61.40 ± 0.11^b^	3.10 ± 0.02^b^	490 ± 2^c^	8.5 ± 0.02^a^	20 ± 0.11e

Means with different superscripts within a column are significantly different (*P* ≤ 0.05).

*Note*: WF is wheat flour bread, WOHQF‐10 is 10% optimal condition treated quinoa flour‐wheat flour, WOHQF‐20 is 20% optimal condition treated quinoa flour‐wheat flour, WOHQF‐30 is 30% optimal condition treated quinoa flour‐wheat flour. WQF‐10 is 10% untreated quinoa flour‐wheat flour, WQF‐20 is 20% untreated quinoa flour‐wheat flour, and WQF‐30 is 30% untreated quinoa flour‐wheat flour.

Abbreviations: DDT, dough development time; DS, dough stability; MC, maximum consistency; MTI, mixing tolerance index; WA, water absorption capacity.

#### 3.4.2 Extensograph Properties

**FIGURE 2:**
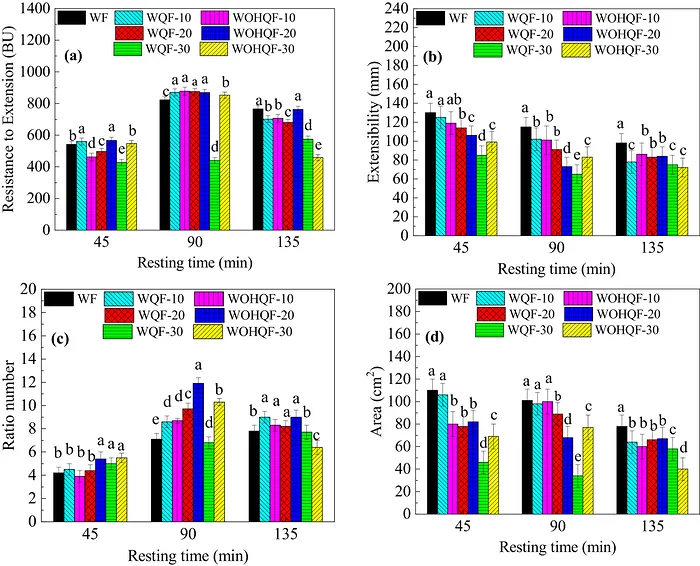
Effect of different substitution levels of optimal hydrothermal treated quinoa flour on the extensograph properties of wheat flour. *Note*: WF is wheat flour bread, WOHQF‐10 is 10% optimal condition treated quinoa flour‐wheat flour, WOHQF‐20 is 20% optimal condition treated quinoa flour‐wheat flour, WOHQF‐30 is 30% optimal condition treated quinoa flour‐wheat flour. WQF‐10 is 10% untreated quinoa flour‐wheat flour, WQF‐20 is 20% untreated quinoa flour‐wheat flour and WQF‐30 is 30% untreated quinoa flour‐wheat flour.

Inclusion of native and hydrothermally treated quinoa flour induced contrasting effects on the extensograph properties of wheat flour (Figure 2). The resistance to extensibility of wheat flour dough showed an increase at resting times higher than 45 min on inclusion of quinoa flour. This was most prominent at 90 min resting time, with 10% optimal hydrothermal‐treated flour inclusion having a 7% higher resistance to extensibility than wheat flour. The increase in resistance to extensibility of wheat dough on inclusion of treated quinoa suggests enhanced gluten‐starch networking within the composite system, which enhanced dough elasticity and strength (Siddique et al. 2024). The extensibility of wheat flour dough showed a decreasing trend with an increase in resting time and quinoa flour inclusion. All treated flours were unable to increase the extensibility of wheat flour dough, with the native quinoa flour having less decline than the treated flour at all levels except at 30% inclusion. This indicates that despite the increase in elasticity, quinoa flour decreased the stretching capacity of wheat dough (Rumler et al. 2022). The ratio number, which is the ratio of resistance to extensibility of wheat flour, showed a similar trend to the resistance to extension results, indicating that the latter played a dominant role. The ratio number of wheat flour dough increased at resting times higher than 45 min on inclusion of quinoa flour. At 90 min, the optimal hydrothermally treated flour at all levels significantly increased the ratio number of wheat flour to a level higher than that of the native flour. Furthermore, there was a decline in the dough area of wheat flour with increasing resting time and quinoa flour inclusion. In general, optimal hydrothermal treatment was unable to alter this trend. This indicates that the energy requirement for deforming wheat flour dough was minimized by quinoa flour inclusion, resting time, and the optimal treatment. Nevertheless, at 90 min, 10% of the optimal hydrothermally treated quinoa flour did not affect the area of the wheat flour dough.

### 3.5 Bread‐Making Functionality of Hydrothermal Treated Quinoa Flour in Wheat Flour Matrix

#### 3.5.1 Bread Quality Evaluation

**FIGURE 3:**
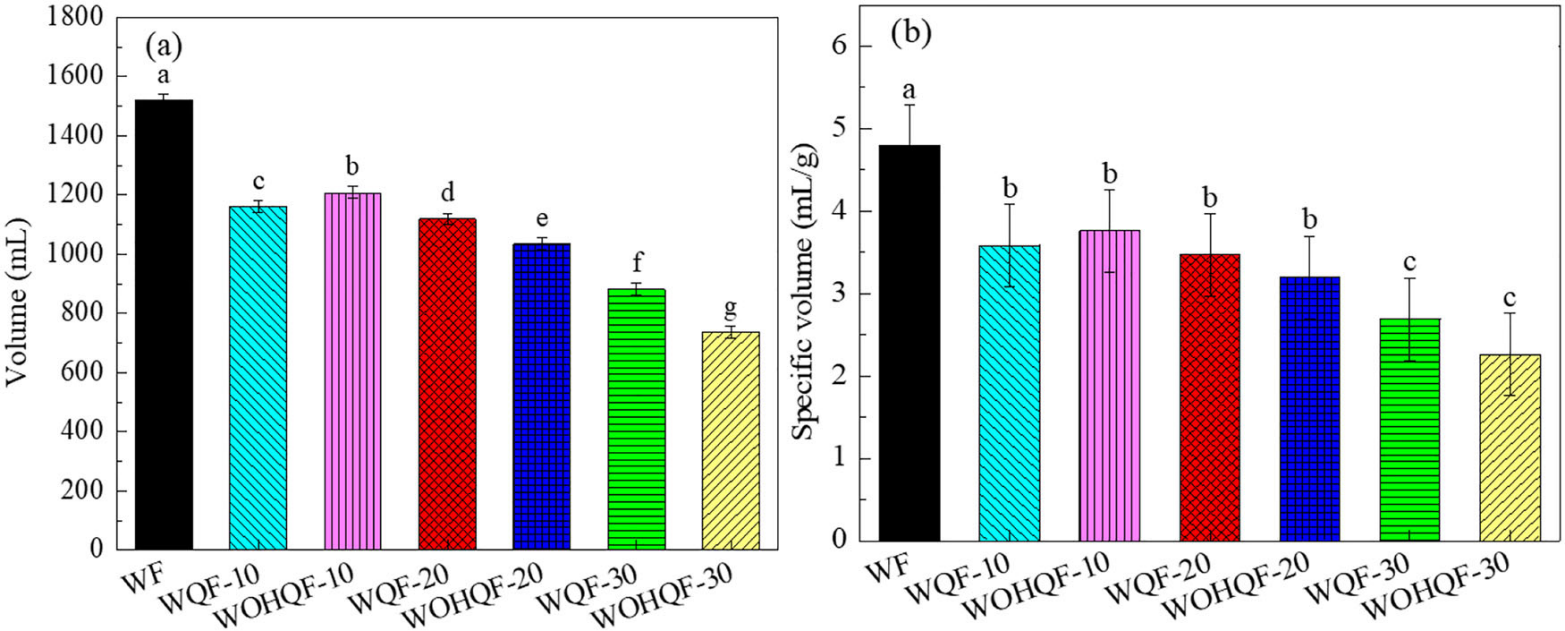
Volume and specific volume of wheat flour bread compared with wheat flour‐optimal hydrothermally treated and untreated quinoa flour breads. *Note*: WF is wheat flour bread, WOHQF‐10 is 10% optimal condition treated quinoa flour‐wheat flour bread, WOHQF‐20 is 20% optimal condition treated quinoa flour‐wheat flour bread, WOHQF‐30 is 30% optimal condition treated quinoa flour‐wheat flour bread, WQF‐10 is 10% untreated quinoa flour‐wheat flour bread, WQF‐20 is 20% untreated quinoa flour‐wheat flour and WQF‐30 is 30% untreated quinoa flour‐wheat flour bread.

The impact of incorporating quinoa flour (WQF) and optimized quinoa flour (WOHQF) at substitution levels of 10%, 20%, and 30% on the loaf volume and specific volume of wheat‐based breads was examined to evaluate their influence on bread structure and baking performance (Figure 3). The wheat bread (demonstrated the highest loaf volume and specific volume, establishing a baseline for comparison. Among the composite breads, those made with 10% WOHQF achieved the highest volume and specific volume values relative to other composite formulations. These breads exhibited good expansion and aeration, suggesting that the optimal heat treatment applied to the quinoa flour contributed to better gas retention and dough development. As the substitution level of WOHQF increased to 20% and 30%, both loaf volume and specific volume showed a gradual decline. Despite the reduction, the 20% WOHQF bread maintained acceptable volume characteristics, while the 30% WOHQF bread demonstrated a visibly denser structure and lower expansion during baking. In comparison, breads containing WQF showed a more pronounced decline in both volume and specific volume across all substitution levels. The superior loaf volume obtained at 10% OHQF substitution can be linked to the improved water absorption capacity and dough stability of the treated flour (Table 5). Enhanced hydration facilitates starch gelatinization and gluten development during baking, improving gas retention within the dough matrix. However, increasing OHQF inclusion beyond 10% progressively diluted the wheat gluten network, thereby reducing its ability to retain fermentation gases. Although hydrothermal treatment improved the compatibility of quinoa flour with wheat flour, the absence of gluten in quinoa ultimately limited loaf expansion at higher substitution levels. These findings demonstrate how modifications in flour functionality and rheology directly translated into bread quality outcomes.

The 30% WQF bread exhibited the lowest volume and specific volume indicating reduced gas‐holding capacity and a tighter crumb structure. Overall, bread formulated with WOHQF outperformed those with WQF in both volume and specific volume, particularly at 10% and 20% substitution levels. These results highlight the beneficial effect of hydrothermal treatment on the functional properties of quinoa flour when used in breadmaking. However, at higher inclusion levels (30%), both types of quinoa flour adversely affected loaf volume, suggesting a threshold beyond which structural quality is compromised. The next section discusses the effect of condition treatment on the sensory properties of quinoa flour in wheat flour matrix.

#### 3.5.2 Sensory Properties

**FIGURE 4:**
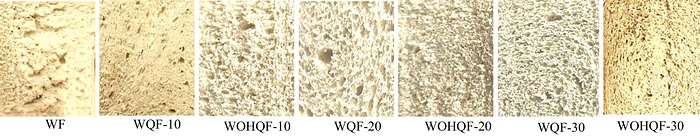
Cross section of crumb grain images of breads from wheat flour and optimized hydrothermally treated and untreated quinoa flour. *Note*: WF is wheat flour bread, WOHQF‐10 is 10% optimal condition treated quinoa flour‐wheat flour bread, WOHQF‐20 is 20% optimal condition treated quinoa flour‐wheat flour bread, WOHQF‐30 is 30% optimal condition treated quinoa flour‐wheat flour bread, WQF‐10 is 10% untreated quinoa flour‐wheat flour bread, WQF‐20 is 20% untreated quinoa flour‐wheat flour and WQF‐30 is 30% untreated quinoa flour‐wheat flour bread.

**FIGURE 5:**
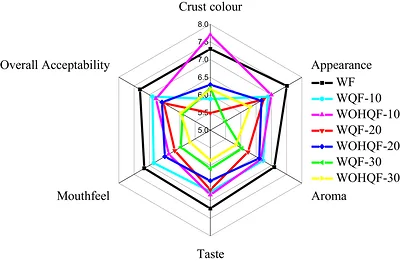
Sensory properties of wheat flour bread and breads containing native or optimal hydrothermal treated quinoa flour. *Note*: WF is wheat flour bread, WOHQF‐10 is 10% optimal condition treated quinoa flour‐wheat flour bread, WOHQF‐20 is 20% optimal condition treated quinoa flour‐wheat flour bread, WOHQF‐30 is 30% optimal condition treated quinoa flour‐wheat flour bread, WQF‐10 is 10% untreated quinoa flour‐wheat flour bread, WQF‐20 is 20% untreated quinoa flour‐wheat flour and WQF‐30 is 30% untreated quinoa flour‐wheat flour bread.

The sensory properties of foods provide valuable information critical to product development and acceptability of the food. Resulting images of the breadcrumb grains from wheat flour breads consisting of untreated and optimal conditioning treated quinoa flours are shown in Figure 4. It was revealed that increase in optimal condition treated quinoa flour inclusion led to more denser bread crumb structures when compared to bread containing native quinoa flour. Notably, bread containing 10% optimal condition treated quinoa flour exhibited a more open crumb structure than its native counterpart. In this study, the effect of untreated and modified quinoa flour on the sensory acceptability of wheat flour bread was studied and is presented as a spider plot (Figure 5). Based on the multiple comparison test analysis, bread containing 10% optimal condition treated quinoa flour exhibited a sensory profile comparable to wheat flour bread, particularly in terms of crust color and appearance. The improved sensory acceptance observed for bread containing 10% OHQF may be attributed to the enhanced hydration characteristics and dough development behavior imparted by hydrothermal treatment (Table 5). The increased water absorption, dough stability, and resistance to extension observed in the composite dough likely promoted more effective gas retention during proofing and baking, resulting in a more open and uniform crumb structure. This was reflected in the crumb grain images and higher loaf volume of the 10% OHQF bread relative to breads containing untreated quinoa flour (Figure 4). In addition, hydrothermal treatment may have improved starch‐protein interactions within the dough matrix, contributing to better crumb formation and a more desirable appearance and mouthfeel. Conversely, increasing OHQF substitution to 20% and 30% produced progressively denser crumb structures and lower loaf volumes, largely due to dilution of the wheat gluten network and reduced gas‐holding capacity (Figure 4). These structural changes likely contributed to lower sensory scores for texture and overall acceptability at higher substitution levels.

## 4 Conclusion

This research work systematically established optimized hydrothermal conditions (12 h conditioning time and 50°C drying temperature), which reduced the retrogradation tendency of quinoa flour. Drying temperature during the treatment had the most impact on the functional properties of quinoa flour. The optimal conditions improved hydration characteristics and reduced retrogradation tendency, although they reduced paste stability and peak viscosity of quinoa flour. This further led to a decline in the volume properties of wheat bread when the optimized treated flour was incorporated. Moreover, the optimized treated flour elicited contrasting effects on the flour rheological properties of wheat flour; however, it was inferred that a 90 min proofing time could adequately produce composite bread dough with optimal extensograph properties. Overall, 10% inclusion of the optimized hydrothermally treated quinoa flour elicited bread properties closest to 100% wheat flour bread in terms of volume and crumb structure, as well as crust color and appearance. It is recommended that further studies build on the outcomes of this study by synergizing the derived optimized hydrothermal treatment conditions with other flour modification methods with the aim of enhancing the swelling/expansion ability as well as the thermal stability of quinoa flour. This study has further highlighted the potential of quinoa as a partial substitute for wheat, which will be advantageous in enhancing food security.

## Author Contributions

**Olayemi E. Dudu**: conceptualization, investigation, writing – original draft, methodology, visualization, validation, writing – review and editing, software, formal analysis, project administration, data curation, and supervision. **Olawunmi O. Bolaji**: investigation, writing – original draft, methodology, validation, writing – review and editing, and formal analysis. **Taiwo A. Olotu**: conceptualization, investigation, writing – original draft, methodology, writing – review and editing, formal analysis, and data curation. **Rukayat A. Tajudeen**: conceptualization, investigation, methodology, validation, data curation, formal analysis, writing – review and editing. **Jessica Wemimo Dudu**: conceptualization, investigation, writing – original draft, methodology, writing – review and editing, formal analysis, project administration, and data curation. **Samson A. Oyeyinka**: conceptualization, methodology, validation, visualization, writing – review and editing, project administration, supervision, and formal analysis.

## Ethics Statement

The sensory evaluation protocol was reviewed and approved by the Departmental Research and Ethics Committee of the Department of Food Science and Technology, Bells University of Technology, Ota, Ogun State, Nigeria. At the time the study was conducted, the committee did not assign reference numbers for ethics approval to approved research projects. All participants were informed about the purpose and nature of the study, and informed consent was obtained prior to participation. Ethical approval of the study has been confirmed through official documentation provided by the department.

## Conflicts of Interest

The authors declare no conflicts of interest.

## Supporting information




**Supplementary Material**: jfds71508‐sup‐0001‐SuppMat.docx
