# Supplementary material for: Optimized Hydrothermal Pretreatment of Quinoa Seeds and Its Impact on the Functional, Rheological, and Breadmaking Properties of Quinoa Flour
Source: J Food Sci. 2026 Sep 25;91(9):e71508. doi: 10.1111/1750-3841.71508 (PMC13613136; doi:10.1111/1750-3841.71508)
Supplement: Supplementary file 1 [file JFDS-91-0-s001.docx]

|  |  |
| --- | --- |
| (a) | (b) |
|  |  |
| (c) | (d) |
|  |  |
| (e) | (f) |
|  | |
| (g) | |

Figure S1. Farinograph curves of the wheat-untreated/optimal hydrothermal treated quinoa flours (a)WQF-10, (b) WOHQF-10, (c)WQF-20, (d) WOHQF-30, (e)WQF-30, (f) WOHQF-30 and (g) WF
